# Authors’ Reply: Equity-Centered Optimization of Virtual Cancer Survivorship Care

**DOI:** 10.2196/79461

**Published:** 2025-08-12

**Authors:** Jacqueline L Bender, Sarah Scruton, Geoff Wong, Stephanie Babinski, Lauren R Squires, Alejandro Berlin, Julie Easley, Sharon McGee, Ken Noel, Danielle Rodin, Jonathan Sussman, Robin Urquhart

**Affiliations:** 1 Cancer Rehabilitation and Survivorship Program, Department of Supportive Care, University Health Network, 585 University Ave, Toronto, M5G 2N2, Canada, 1 416-581-8606; 2 Dalla Lana School of Public Health, University of Toronto, Toronto, ON, Canada; 3 Nuffield Department of Primary Care Health Sciences, University of Oxford, Oxford, United Kingdom; 4 Radiation Medicine Program, Princess Margaret Cancer Centre, University Health Network, Toronto, ON, Canada; 5 Department of Radiation Oncology, University of Toronto, Toronto, ON, Canada; 6 Department of Medical Education, Dr Everett Chalmers Regional Hospital, Horizon Health Network, Fredricton, Canada; 7 Department of Medicine, Division of Medical Oncology, The Ottawa Hospital and the University of Ottawa, Ottawa, ON, Canada; 8 The Walnut Foundation, Brampton, ON, Canada; 9 Department of Oncology, McMaster University, Hamilton, Canada; 10 Department of Community Health and Epidemiology, Dalhousie University, Halifax, Canada

**Keywords:** cancer, follow-up, virtual, outcomes, realist evaluation, survivorship

We thank Bhatti and Bhatti [1] for their positive feedback and agree with their recommendations to incorporate policy-level contextual factors in future iterations of the virtual follow-up (VFU) program theory.

Participants’ discussion of systemic and structural factors influencing VFU use was limited, likely due to the sociodemographic makeup of the sample [2]. Although we purposively selected for and achieved diversity in race, ethnicity, country of origin, spoken language, and gender, most participants were highly educated, activated patients living in high-income households. Hence, we acknowledged that the findings may not represent the perspectives of less activated, less educated patients who may have lower health literacy, are less involved in their care, and face more challenges with accessing virtual care. Thus, we concluded that further research is needed to better understand how VFU could be optimized for individuals who face systemic and structural barriers to care. We agree that this would involve tailored and targeted strategies for recruiting structurally marginalized individuals to test context-mechanism-outcome configurations in varied contexts.

Despite their higher socioeconomic status, participants emphasized the critical importance of ensuring that VFU technology is accessible, easy to use, and reliable [2]. We agree that key policy-level strategies for overcoming these structural barriers to VFU include equitable VFU reimbursement structures for health care professionals and reliable and affordable broadband access for patients. As we explained in the paper, telephone visits must be sustained to bridge the digital divide, along with changes to physician reimbursement structures to ensure equitable compensation for such visits. In parallel, as Bender et al [3] stated in a prior article where they demonstrated the critical importance of broadband as a determinant of health-related internet use, we believe that reliable and affordable broadband must be a priority. Not having broadband considerably limits one’s internet quality and access to essential services, leading some researchers to suggest that broadband access is a social determinant of health [4]. In 2020, the Government of Canada invested CAD $3.2 billion (US $2.3 billion) to provide all Canadians with high-speed internet and mobile cellular access by 2030. Future work should assess the impact of this policy on VFU.

It was encouraging to learn that newer studies are also advocating for empathy training in telehealth curricula to optimize VFU for patients [5]. We agree that telehealth training should be guided by antiracist, decolonial, and trauma-informed frameworks to address disparities in emotional support quality during VFU. However, we recommend equity, diversity, and inclusion training for recognizing and addressing unconscious bias, communicating empathy, and promoting cultural safety in a manner tailored to each visit type, given that structurally marginalized patients are less satisfied with their care regardless of visit type [6].

In summary, we thank Bhatti and Bhatti [1] for their timely recommendations on strengthening equity-centered implementation of telehealth in survivorship care. This constructive feedback will be incorporated into a joint Multinational Association of Supportive Care in Cancer (MASCC)/American Society of Clinical Oncology (ASCO) update of the ASCO Telehealth in Oncology Standards, which will include considerations for resource-constrained settings [7].

## Abbreviations

ASCO: American Society of Clinical Oncology
MASCC: Multinational Association of Supportive Care in Cancer
VFU: virtual follow-up
